# A Review of Systemic Biologics and Local Immunosuppressive Medications in Uveitis

**DOI:** 10.18502/jovr.v17i2.10804

**Published:** 2022-04-29

**Authors:** Neesurg S. Mehta, Parisa Emami-Naeini

**Affiliations:** ^1^Department of Ophthalmology and Vision Science, University of California, Davis, Sacramento, CA, USA

**Keywords:** Corticosteroids, Immunosuppression, Uveitis

## Abstract

Uveitis is one of the most common causes of vision loss and blindness worldwide. Local and/or systemic immunosuppression is often required to treat ocular inflammation in noninfectious uveitis. An understanding of safety and efficacy of these medications is required to individualize treatment to each patient to ensure compliance and achieve the best outcome. In this article, we reviewed the effectiveness of systemic biologic response modifiers and local treatments commonly used in the management of patients with noninfectious uveitis.

##  INTRODUCTION

Uveitis, defined as inflammation in the uveal tract, is a major cause of vision loss and disability especially in the young working-age population.^[1]^ Based on the etiology of inflammation, it can be categorized into infectious or noninfectious uveitis (NIU). The mainstay of treatment in NIU is systemic and/or local immunosuppression.

Systemic therapy with corticosteroids or corticosteroid-sparing immunomodulatory therapy (IMTs) is used for long-term control of uveitis. These medications achieve sustained control of inflammation while avoiding the risk of peri- or intraocular corticosteroid injections. However, they are associated with an increased risk of systemic infections, malignancies, and other side effects.^[1,2,3,4]^Local treatment, on the other hand, yields faster control of inflammation without causing systemic immunosuppression. These medications, however, are associated with a higher risk of ocular side effects including infection, increased intraocular pressure (IOP), and cataract formation. Often times, a combination of systemic and local treatment is required in order to achieve appropriate control of inflammation. In thisarticle, we reviewed biologic response modifiers (or biologics) and local treatments used in the management of NIU.

### Systemic Biologics

Immunomodulatory therapy (IMTs) modifies the immune response and controls inflammation. These medications are divided into conventional IMTs and biologics. Conventional IMTs, including antimetabolites (methotrexate, azathioprine, mycophenolate mofetil) are usually the first-line treatment.^[1]^ Other IMTs include calcineurin inhibitors (cyclosporine, sirolimus, tacrolimus) and alkylating agents (cyclophosphamide, chlorambucil).^[1]^ Biologics are used when NIU is recalcitrant to conventional IMTs and as first-line treatment in select cases.

### Tumor necrosis factor-a inhibitors

These agents constitute a group of biologics that suppress TNF-a, which is an integral cytokine in the inflammatory cascade.^[1]^ Currently, five TNF-a inhibitors are approved by the United States Food and Drug Administration (FDA) for the treatment of rheumatologic conditions, which include infliximab, adalimumab, golimumab, and certolizumab, all of which are monoclonal TNF-a antibodies, in addition to etanercept, which is a fusion protein that functions as a decoy receptor that binds to TNF-a.^[5]^

Common side effects of this group of medications include headache, nausea, abdominal pain, diarrhea, constipation, with more severe complications including cytopenia, hepatotoxicity, heart failure, malignancy, and reactivation of infections.^[6]^ Higher risk of demyelinating disorders has been linked to these medications. Hence, they should not be used in patients with history of multiple sclerosis and used with caution if a family history of demyelinating disease is present.^[7,8]^ Anti-TNF-a induced lupus is another complication that is most commonly seen with infliximab use, followed by etanercept and adalimumab.^[9]^ The mechanism is proposed to be autoantibody production secondary to a “cytokine shift" due to anti-TNF-a suppression of T helper 1 response, and hence increasing T helper 2-mediated cytokine and autoantibody production.^[9]^ Treatments involve discontinuation of the anti-TNF-a agents, and sometimes addition of immunosuppressants.^[9]^

#### Infliximab 

Infliximab (RemicadeⓇ) is an IgG1 chimeric monoclonal antibody against membrane-bound and soluble TNF-a and is administered as an intravenous infusion most commonly at weeks 0, 2, and 6 (induction), and then every eight weeks (maintenance).^[10]^ In uveitis patients, infliximab may be given at a low ( < 10 mg/kg), moderate ( ≥ 10–15 mg/kg), or a high ( ≥ 15–20 mg/kg) dose.^[11]^ Efficacy of infliximab has been widely studied in treatment of uveitis associated with Behcet's disease (BD),^[12,13,14,15,16,17]^ juvenile idiopathic arthritis (JIA),^[18,19,20,21,22]^ sarcoidosis,^[23]^ and other NIUs. These studies have shown an overall clinical efficacy and remission rate of 80–90% for infliximab.^[24,25,26]^

In a prospective observational study looking at patients with refractory posterior uveitis receiving infliximab, a complete response was noted in 68% of patients.^[27]^ Another study noted a complete remission in 40% of patients after only three infusions.^[28]^ A retrospective study looking at long-term ( > 2 years) use of infliximab in patients with refractory JIA-associated uveitis showed clinical remission (defined as no flares for > 6 months) in ∼ 20% of patients,^[22]^ while another study looking at pediatric patients with refractory NIU noted uveitis control in 89% of patients.^[20]^ In a meta-analysis evaluating efficacy of anti-TNF-a therapy in childhood uveitis, infliximab was found to be effective in 72% of patients.^[21]^

Response to infliximab (and less commonly, other anti-TNF-a agents) can be adversely affected by the presence of anti-TNF-a antibodies. These antibodies are reportedly seen in ∼ 20% of patients and can cause treatment failure and reaction to infliximab infusions.^[29]^ Concurrent use of immunosuppressant including methotrexate or azathioprine can decrease the risk of autoantibody formation by 50%.^[29]^ Autoantibodies are less commonly seen in adalimumab, which is due to the fact that adalimumab is a fully humanized molecule.^[9]^

#### Adalimumab

Adalimumab (HumiraⓇ) is a fully humanized anti-TNF-a monoclonal antibody and is the only IMT approved by the FDA for use in patients with intermediate, posterior, or panuveitis.^[30]^ It is administered subcutaneously 40 mg (or 20 mg if patient's weight is < 30 kg) every other week, often after an initial loading dose of 80 mg.^[30]^ It has been commonly used to treat uveitis associated with JIA,^[18,21,22,31,32]^ sarcoidosis,^[33]^ ankylosing spondylitis (AS),^[34]^ and BD,^[35]^ in addition to other NIUs.^[36,37,38,39,40,41]^

In a double-blind, randomized, placebo-controlled trial, the SYCAMORE study group looked at patients on a stable dose of methotrexate with active JIA-associated uveitis and found that the addition of adalimumab resulted in lower rates of treatment failure (27% in the treatment group vs 60% in the control group, *P* < 0.0001).^[31]^ The Visual I Trial, a phase-3 randomized clinical trial, showed that use of adalimumab was associated with a lower rate of uveitis flare and treatment failure in patients with active NIU.^[37]^ The VISUAL II Trial was conducted to assess efficacy of adalimumab in inactive NIU and found that treatment failure occurred in 39% of patients treated with adalimumab compared to 55% in the control group, and time to treatment failure was longer in the adalimumab group.^[40]^ The VISUAL III trial was a phase-3 open-label extension study involving patients who met treatment failure criteria or those who completed the VISUAL I and II trials and were followed to 78 weeks.^[41]^ This study found that 60% of patients with active uveitis and 74% of patients with inactive uveitis achieved quiescence on adalimumab.^[41]^

Although there are no randomized clinical trials comparing the efficacy of infliximab and adalimumab in uveitis, there are many retrospective and observational studies juxtaposing the two anti-TNF-a agents. While several studies have reported similar efficacy between the two drugs in treatment of NIU,^[42,43,44]^ some studies evaluating JIA-associated uveitis have noted better efficacy of adalimumab compared to infliximab.^[18,21,22]^ Drug retention rates were similar between the two groups and concomitant use of disease-modifying antirheumatic drugs or treatment history did not affect the retention rates.^[45]^ It has also been reported that in the case of loss of initial response of uveitis to one of these two agents, switching to the other one may result in better control of inflammation.^[46]^

#### Golimumab 

Golimumab (SimponiⓇ), a fully human anti-TNF-a monoclonal antibody, is administered monthly (50 mg) as a subcutaneous injection^[47]^ and has been studied in uveitis associated with spondyloarthropathies,^[48,49]^ BD,^[50,51]^ and other NIUs.^[52,53]^ The GO-EASY study looked at patients treated with Golimumab for AS-related uveitis and noted a reduction in acute anterior uveitis rate.^[49]^

#### Certolizumab pegol 

Certolizumab (CimziaⓇ) is pegylated Fab fragment of humanized monoclonal antibody against TNF-a.^[56]^ It is administered subcutaneously every other week for three injections (400 mg/dose) initially, and then 200 mg every other week.^[54]^ It has been studied most commonly in uveitis associated with spondyloarthropathy, in addition to other refractory NIUs.^[55,56,57,58]^ In an ongoing 96-week open-label phase-4 study, the 48 weeks results revealed an 87% reduction in the incidence of anterior uveitis flare in patients with spondyloarthropathy associated anterior uveitis.^[59]^ Similarly, the RAPID-axSpA trial showed a decrease in the frequency of uveitis flares over 96 weeks.^[54]^

#### Etanercept

Etanercept (EnbrelⓇ) functions as a decoy receptor for TNF-a.^[60]^ Although effective in treating rheumatologic conditions, it is not commonly used for uveitis as it is not as efficacious in comparison to other anti-TNF-a agents,^[60]^ and there is risk for drug-induced uveitis and sarcoidosis.^[61,62]^

### Anti-interleukin-1 

*Anakinra and Canakinumab*

Anakinra (KineretⓇ) is a humanized IL-1 receptor antagonist and Canakinumab (IlarisⓇ) is an anti-IL-1B monoclonal antibody.^[63,64]^ They have shown effectiveness in uveitis associated with BD and MS.^[65,57,68]^ Adverse reactions to this group of medications include injection site reactions, anaphylaxis, and infections such as pneumonia.^[69]^

*Gevokizumab*

Gevokizumab (Xoma 052) is a humanized anti-IL-1β monoclonal antibody and is administered intravenously or subcutaneously.^[70,71,72]^ It has been studied in uveitis associated with BD and noninfectious scleritis.^[62][63,64][73]^ Although rapid control of BD-related uveitis was noted in the pilot and phase-2 studies,^[70,71]^ the phase-3 EYEGUARD-B trial failed to meet its primary endpoint and the medicine did not significantly alter the risk of flares.^[72]^

### Anti-interleukin-2

#### Daclizumab

Daclizumab (ZinbrytaⓇ) is a humanized monoclonal antibody that inactivates T lymphocytes by binding to the CD25 portion of the IL-2 receptor and is administered subcutaneously.^[74]^ Nussenblatt et al. reported that patients with NIU treated with Daclizumab needed less concomitant immunosuppression while maintaining baseline vision at 26 weeks.^[75]^ However, the medicine was withdrawn from the market in 2018 following cases of hepatic injury and meningoencephalitis.^[76]^

### Anti-IL-interleukin-6

#### Tocilizumab

Tocilizumab (ActemraⓇ) is a recombinant humanized monoclonal antibody against the IL-6 receptors and is most commonly administered subcutaneously (162 mg every two weeks).^[79]^ An intravenous form is also available. Efficacy of this medicine has been studied in uveitis associated with JIA,^[77,78]^ BD,^[79]^ uveitic macular edema,^[80,81,82,83]^and other NIUs. Calvo-Rio et al^[78]^ found that tocilizumab was effective in treating refractory JIA-associated uveitis, however, the phase-2 APTITUDE trial noted efficacy of only 34% which did not meet the primary endpoint of control of inflammation at week 12.^[77]^ In the STOP-Uveitis study, 37 patients received monthly intravenous infusions of 4 mg/kg or 8 mg/kg of tocilizumab over six months and found that 43% of patients had two-step decrease in vitreous haze and 30% gained two lines or more vision, although there was no statistically significant difference in vision and central macular thickness (CMT) between two doses.^[84]^ Common side effects include injection site reactions, arthralgia, and headaches.^[77]^

#### Sarilumab

Sarilumab (KevzaraⓇ) is a human monoclonal antibody blocking the IL-6 receptor and is commonly administered subcutaneously.^[85]^ The phase-2 SATURN study evaluated the efficacy and safety of Sarilumab over 16 weeks in patients with intermediate, posterior, or panuveitis.^[85]^ This study found that in comparison to the placebo group, patients taking Sarilumab achieved a statistically significant reduction in use of corticosteroids or at least two-step reduction in vitreous haze when assessed by investigators (64.0% vs 35.0%, *P* = 0.03), but not when assessed based on fundus photography and by central reading center (46.1 vs 30.0%, *P* = 0.2).^[85]^ The most common adverse reactions include injection site reaction, respiratory tract infection, urinary infections, nasopharyngitis, transaminitis, while more severe adverse reactions include neutropenia and infections.^[86]^

### Anti-interleukin-17

Secukinumab (CosentyxⓇ) is a fully human monoclonal antibody against IL-17A and is administered subcutaneously.^[87]^ Three phase-III trials examined the efficacy of Secukinumab in patients with BD-associated uveitis, active NIU, and quiescent NIU.^[87]^ None of these studies showed a statistically significant difference in uveitis recurrence between Secukinumab and placebo. The most common adverse reactions include headache, diarrhea, upper respiratory infections, neutropenia, inflammatory bowel disease, and malignancy.^[88]^

### Anti-interleukin-23

Ustekinumab (StelaraⓇ) is a humanized monoclonal antibody against IL-12 and IL-23 and is administered most commonly subcutaneously.^[89]^ Studies have reported successful control of uveitis associated with psoriatic arthritis, plaque psoriasis,^[90]^ and Crohn's disease.^[91]^ Adverse reactions include headache, fatigue, injection site reaction, upper respiratory infections, urinary tract infections, malignancy, and cardiovascular events.^[89]^

### Anti-CD20 

Ritixumab (RituxanⓇ) is a humanized monoclonal antibody against CD20 and is administered intravenously.^[92,93,94,95,96]^ It has been studied in uveitis associated with BD,^[96,97]^ JIA,^[93,98]^ noninfectious scleritis,^[99,100,101]^ granulomatosis with polyangiitis,^[102,103,104,105,106]^ Vogt-Koyanagi-Harada disease (VKH),^[107]^ Susac syndrome,^[108]^ in addition to other NIUs.^[92,109,110]^ Lasave et al reported on 21 eyes with refractory noninfectious posterior uveitis of which 82% achieved control of inflammation on fluorescein angiography at two years.^[92]^ Cao et al examined patients with refractory noninfectious scleritis and found that 86.6% of cases achieved scleritis activity score of zero at month six.^[99]^ Common adverse reactions include infusion reaction (fever, rigor, and chills), while more severe side effects include infections, progressive multifocal leukoencephalopathy, and hepatitis B reactivation.^[110]^

### Selective costimulation modulator

Abatacept (OrenciaⓇ) is a recombinant fusion protein made up of the extracellular domain of human cytotoxic T-lymphocyte antigen 4 (CTLA-4) and fragment of Fc domain of human IgG.^[111]^ This molecule competitively inhibits antigen-presenting cells (APCs) from binding to CD80 and CD86 on T cells, hence, preventing APCs from delivering a co-stimulatory response and activating T cells.^[111]^ Studies have shown that Abatacept can be effective in controlling JIA-associated uveitis.^[112,113,114,115]^ Common side effects include headache, upper respiratory tract infection, and nausea while serious adverse reactions include infection.^[116,117]^

### Interferons

Interferons have been studied widely in the treatment of NIU,^[118]^ especially in BD-associated uveitis.^[119,120,121,122,123,124,125]^ Studies have found that IFN-alpha is effective in 84–92% of patients with BD-associated posterior or panuveitis^[121,123]^ as well as uveitic macular edema^[126,127]^ and macular edema related to presumed ocular tuberculosis.^[128,129]^ IFN-beta has shown effectiveness in reducing macular edema and improving vision in intermediate uveitis-associated macular edema.^[130]^ Common adverse effects include nausea, fatigue, flu-like symptoms, psychiatric sequelae, elevated transaminases, and hematologic toxicity.^[118,131]^

### Janus kinase inhibitors

Tofacitinib (XeljanzⓇ) is a small molecule that reversibly inhibits Janus-associated kinases (JAKs).^[132]^ JAKs mediate cytokine receptor signaling, initiating a downstream pathway which eventually leads to transcription of inflammatory genes.^[132]^ In a case of JIA-associated uveitis only responsive to intravitreal dexamethasone implants, tofacitinib successfully controlled the uveitis and arthritis, and the patient no longer required corticosteroid implants.^[133]^ Similarly, Misrocchi et al.^[134]^ reported on four cases of refractory JIA-associated uveitis and noted a good uveitis response, though a less favorable arthritis response when treated with JAK inhibitors. Paley et al^[132]^ reported one case of refractory anterior and intermediate uveitis and another case of refractory scleritis treated successfully with JAK inhibitors. Common adverse effects include upper respiratory infection, diarrhea, headache, and malignancy.^[135]^

### Local Treatments

Peri- or intraocular administration of immunosuppressives can provide a high dose of medication to the posterior segment of the eye while avoiding systemic side effects. These medications are more commonly used in unilateral uveitis.

### Periocular or intraocular corticosteroid injections

Local corticosteroids have been widely studied in the treatment of uveitis. These medications are effective at local control of inflammation. Side effects include ocular infection, cataract formation and increase in IOP.^[136]^

#### Triamcinolone acetonide

Triamcinolone acetonide (TA) can be administered adjacent but outside the globe in the posterior sub-Tenon space through the orbital septum (KenalogⓇ 40 mg/ml, Bristol-Myers Squibb Company, Princeton, NJ) or in the vitreous cavity (TriesenceⓇ 40 mg/ml, Alcon Pharmaceuticals, Fort Worth, TX). TriesenceⓇ is preservative-free and more expensive than the preserved formulation (KenalogⓇ). Sub-Tenon's TA typically lasts two to three months, although this can be variable due to variable crystal sizes. Intravitreal TA crystals are more uniformly sized, hence, TriesenceⓇ has a more predictable duration of action of four to six weeks (less in vitrectomized eyes).^[136]^ Effectiveness of these steroids has been studied extensively.^[137]^ It has been demonstrated that both injections are effective in patients with NIU, however, intravitreal corticosteroids were more effective in improving vision and macular edema, although a significantly higher risk of IOP elevation was associated with intravitreal corticosteroids compared to periocular injection.^[137]^

#### Dexamethasone intravitreal implant

The Dexamethasone implant (OzurdexⓇ) is a bioerodible, sustained release injectable implant that gradually releases dexamethasone into the posterior chamber and usually lasts three to four months.^[138]^ It is FDA approved for use in patients with posterior segment inflammation. In a prospective, randomized, controlled clinical trial, the HURON Study group looked at patients with noninfectious intermediate or posterior uveitis who were randomized to receive dexamethasone implant (0.7 mg or 0.35 mg) or sham.^[138]^ At week eight (primary endpoint), patients in the dexamethasone implant group experienced significant reduction in vitreous haze and CMT compared to the control group. The percentage of eyes gaining > 15 letters was two- to sixfolds higher in the implant groups as compared to the sham group. Less than 5% of the eyes developed IOP of > 35 mmHg across all groups. Other side effects included cataract formation (as high as 29% after 12 months) and migration of implant to the anterior chamber, which caused corneal decompensation.^[139]^

#### Fluocinolone acetonide intravitreal implant (injectable)

The fluocinolone acetonide intravitreal implant (YutiqⓇ) (FAi) is a non-bioerodible injectable intravitreal implant containing 0.18 mg fluocinolone acetonide. It is designed to release the steroid over 36 months and is FDA approved for noninfectious posterior uveitis.^[140]^ In a large prospective, randomized, sham-controlled trial, Jaffe et al^[140]^ evaluated 129 patients with chronic noninfectious posterior uveitis over three years who received either the FAi or sham. At 36 months, the recurrence rate was 5.7% in FAi group versus 28.6% in sham group (*P* < 0.001) and the median time to first recurrence was significantly lower in the FAi group (657 days vs 70.5 days, respectively).^[141]^ About 11.9% of the patients in the FAi group required glaucoma surgery (compared to 5.7% in the sham group) and as expected most patients (73.8%) in the FAi group required cataract surgery (compared to 23.8% in the sham group).^[141]^

#### Fluocinolone acetonide implant (surgical)

The Retisert Ⓡ implant contains 0.59 mg fluocinolone acetonide (releasing approximately 2 µg/day for three years) and is implanted through the pars plana in the vitreous cavity during surgery.^[142]^ The Multicenter Uveitis Steroid Treatment (MUST) trial compared this implant to systemic immunosuppression and found that although the implant group showed improved visual acuity and uveitis control at two years,^[142]^ the opposite was true at seven years.^[143]^ This should be interpreted with caution as majority of patients who received the implant did not receive an additional implant and a significant number of patients were lost to follow-up or received cross-over treatment.

#### Suprachoroidal corticosteroids

Administration of drugs in the suprachoroidal space (between choroid and sclera) has been investigated as an alternate technique for targeted delivery of molecules to the posterior chorioretinal structures.^[144]^ An investigational corticosteroid formulation (preservative-free TA, CLS-TA, Clearside Biomedical, Alpharetta, Georgia, USA) injected into the suprachoroidal space has been studied in patients with uveitic macular edema. Safety and efficacy of this formulation was studied in an open label safety trial (AZALEA trial) and phase-3 randomized trial (PEACHTREE trial).^[145,146]^ These studies showed a statistically significant reduction in macular edema and improvement in visual acuity in the treatment arm compared to the control group, without a significant increase in the rate of cataract formation or IOP.^[146]^ This special formulation (Xipere ${}^{TM}$ , TA injectable suspension 40 mg/ml, Bauch + Lomb and Clearside Biomedical) injected via the proprietary MicroinjectorⓇ recently received FDA approval for use in the treatment of uveitis macular edema.

### Non-steroidal injections

#### Methotrexate

Methotrexate was the first systemic non-steroidal IMT that received FDA approval for use in autoimmune disease.^[147]^ Efficacy of intravitreally administered methotrexate in patients with noninfectious uveitis has been reported in retrospective studies.^[148,149]^ Safety and efficacy of this drug in patients with macular edema is currently under investigation in the Macular Edema Ranibizumab versus Intravitreal Anti-inflammatory Therapy (MERIT) multicenter, randomized controlled.^[150]^Intravitreal methotrexate is also used in patients with vitreoretinal lymphoma.^[151]^ Side effects include risk of infection and corneal epitheliopathy.^[151]^

#### Sirolimus

Sirolimus is an inhibitor of the mammalian target of rapamycin and downstream production of proinflammatory cytokines.^[152,153]^ The one-year results from the SAVE1 study showed that although intravitreal formulation was better tolerated, there was no difference in efficacy between subconjunctival and intravitreal administration of Sirolumus in treatment of noninfectious intermediate, posterior or panuveitis.^[153,154]^ The SAVE2 trial illustrated that monthly injections of 440 µg intravitreal Sirolimus was better at reducing vitreous haze, CMT, and improving vision when compared to the 880 µg bimonthly dose.^[155]^ In a combined analysis of 592 patients in phase-3 trials, SAKURA1 and -2, Merrill et al^[152]^ showed that a significantly higher proportion of patients treated with intravitreal Sirolimus 440 µg compared with 44 µg achieved vitreous haze of 0 at five months, though there were similar percentages of patients with > 5 letter improvement in vision and those tapered off corticosteroids in both dose groups.

#### Anti-TNF agents

Efficacy and safety of intravitreal infliximab was studied in animal models and human studies.^[156,157,158,159]^ Human studies reported variable efficacy of intravitreal infliximab in controlling inflammation but raised safety concerns, including development of intraocular inflammatory response,^[156]^ decreased electroretinogram amplitudes, and visual field measurements.^[157]^ Intravitreal adalimumab has shown effectiveness in controlling inflammation and macular edema in small retrospective studies.^[160,161]^ More studies are required to evaluate long-term safety and efficacy of intravitreally administered medicine.

#### Rituximab

Intravitreally administered Rituximab (alone or together with methotrexate) is widely used and proven effective in patients with vitreoretinal lymphoma.^[162]^Side effects include transient IOP elevation and iridocyclitis.^[163]^

### Novel Technologies: Electroporation

The biotechnology company Eyevensys (Paris, France) has created the first gene expression technology that uses plasmids to induce production of cytokines.^[164]^ Using a small needle, the device inserts plasmids into the ciliary muscle and a series of short electrical pulses induce uptake of these plasmids into the cells.^[164]^ EYS606 is a treatment being investigated in an ongoing phase-I/II trial (NCT03308045) in the European Union for treatment of NIU by introducing a plasmid encoding for TNF-a inhibitors. Preliminary study included nine patients receiving escalating doses of the EYE606 treatment.^[165]^ One patient noted a ≥ 10 letter gain in vision after two weeks and two patients noted a ≥ 20% reduction in macular edema and ≥ 12 letter gain in vision after six to eight months (186).^[165]^ The ongoing part two of the study will assess safety and efficacy of the highest and maximally tolerated EYS606 dose over 48 weeks. The Electro Study ((NCT03308045) is an ongoing phase-2 trial in the US assessing safety and efficacy of two doses of EYS606.^[166]^

### Common Practice and Future Horizons

Systemic and/or local immunosuppression are mainstay of treatment in NIU and usually a combination of these modalities is needed to control inflammation and achieve quiescence. In systemic management of uveitis, a “stepladder” approach is recommended. This approach starts with local and/or systemic corticosteroids followed by IMTs (as needed) with the goal of reducing the dose of systemic corticosteroids to < 5–10 mg of prednisone per day.^[167,168]^Patients with chronic uveitis need to be counselled on the need for long-term treatment, the different options available to them, and the side effects of systemic and local therapeutics, all in an effort to tailor treatment to each patient and increase long-term compliance. The advent of novel local and systemic treatment options has decreased the risk of breakthrough inflammation and long-term vision loss in patients with NIU.

##  Financial Support and Sponsorship

Nil.

##  Conflicts of Interest

The authors declare that they have no conflict of interest.
